# Pathophysiology and clinical manifestations of immune complex vasculitides

**DOI:** 10.3389/fmed.2023.1103065

**Published:** 2023-03-03

**Authors:** Cord Sunderkötter, Linda Golle, Evangéline Pillebout, Christiane Michl

**Affiliations:** ^1^Department of Dermatology and Venereology, University Hospital Halle, Martin-Luther-University Halle-Wittenberg, Halle, Germany; ^2^Laboratory Nephrology Unit, Saint Louis Hospital, INSERM 1149, CRI, Paris, France

**Keywords:** IgA vasculitis, cryoglobulinemic vasculitis, rheumatoid vasculitis, hypocomplementaemic vasculitis, serum sickness, glomerulonephritis IgA1, cutaneous IgM/IgG-vasculitis, immune complex disease

## Abstract

Immune complex (IC) vasculitides present inflammations of vessel walls associated with perivascular deposition of immunoglobulins (Igs), mostly ICs. They encompass systemic and skin-limited variants of IgA vasculitis (IgAV), cryoglobulinemic vasculitis (CV), rheumatoid, lupus, and hypocomplementemic vasculitides, serum sickness cutaneous IgM/IgG (non-IgA) vasculitis, and recurrent macular (hypergammaglobulinemic or exertion-induced) vasculitis. Serum sickness and CV fulfill the criteria of a type III hypersensitivity immune reaction as large lattices of the IC precipitate at vessel walls and activate polymorphonuclear neutrophils (PMNs). Immunoglobulin-A vasculitis differs with regard to the causes of perivascular deposition of ICs since here many IgA1 molecules are hypoglycosylated (Gd-IgA1), which appears to facilitate their perivascular deposition in skin and mesangium (via e.g. CD71). The reasons for increased generation of immunoglobulins or formation of IC and their perivascular deposition in either skin or systemic organs are different and not fully explored. A common denominator of OC vasculitides is the activation of PMNs near the vessel wall *via* Fcy or Fcα receptors. Acute episodes of IgAV additionally require PMNs to become preactivated by IgA1 or by IC already in circulation. This intravascular priming results in increased adherence and subsequently vessel-destructive NETosis when they encounter IgA deposited at the vessel walls. Binding of IgA1 to PMNs in blood stream is associated with increased serum levels of hypogalactosidated IgA1. The characteristic clinical picture of IgAV (and also of so-called IgG/IgM vasculitis) comprises palpable or retiform purpura with a clear predilection for lower legs, probably due to stasis-related reduction in blood velocity, while in other IC vasculitides, additional factors influence the sites of vasculitides. Our knowledge of distinct forms and different pathophysiological pathways of IC vasculitides may lead to in efficacious or targeted therapies. Antibodies to complement components or intestinal budesonide for IgAV are promising agents (the latter suppresses the pathophysiologically related IgA nephropathy by reducing the generation of mucosal IgA.

## 1. Definition and different forms of immune complex vasculitides

Immune complex vasculitides present inflammations of vessel walls associated with and mainly elicited by perivascular deposition of immunoglobulins, mostly in the form of immune complexes (ICs).

**Figure 1:**
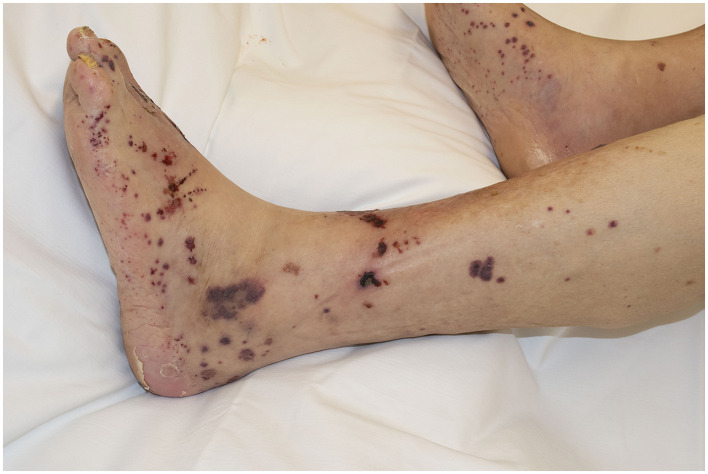
Round or oval and retiform (or branched) palpable purpura in IgA vasculitis.

**Figure 2:**
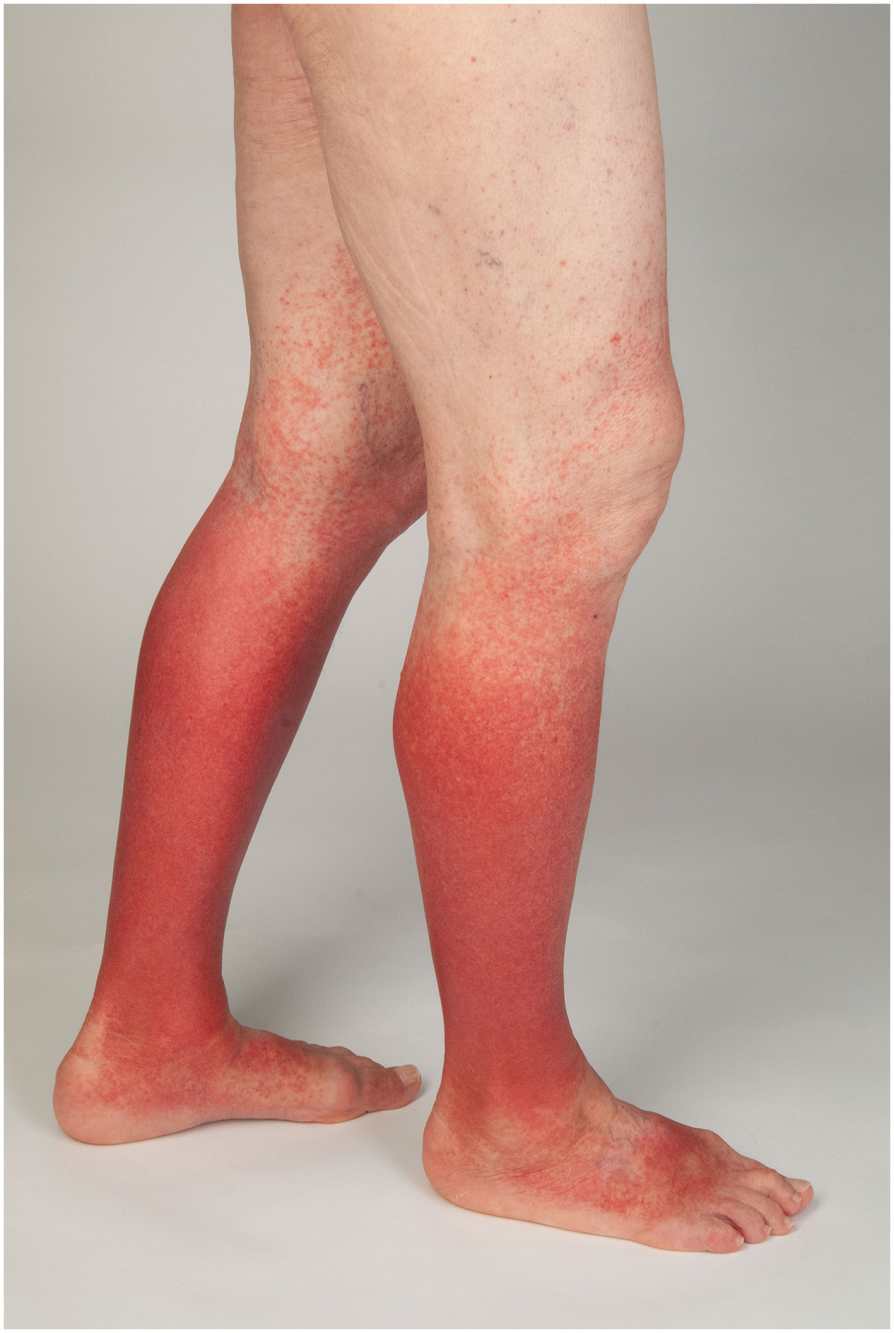
Cutaneous hypocomplementemic IgG/IgM-vasculitis.

**Figure 3:**
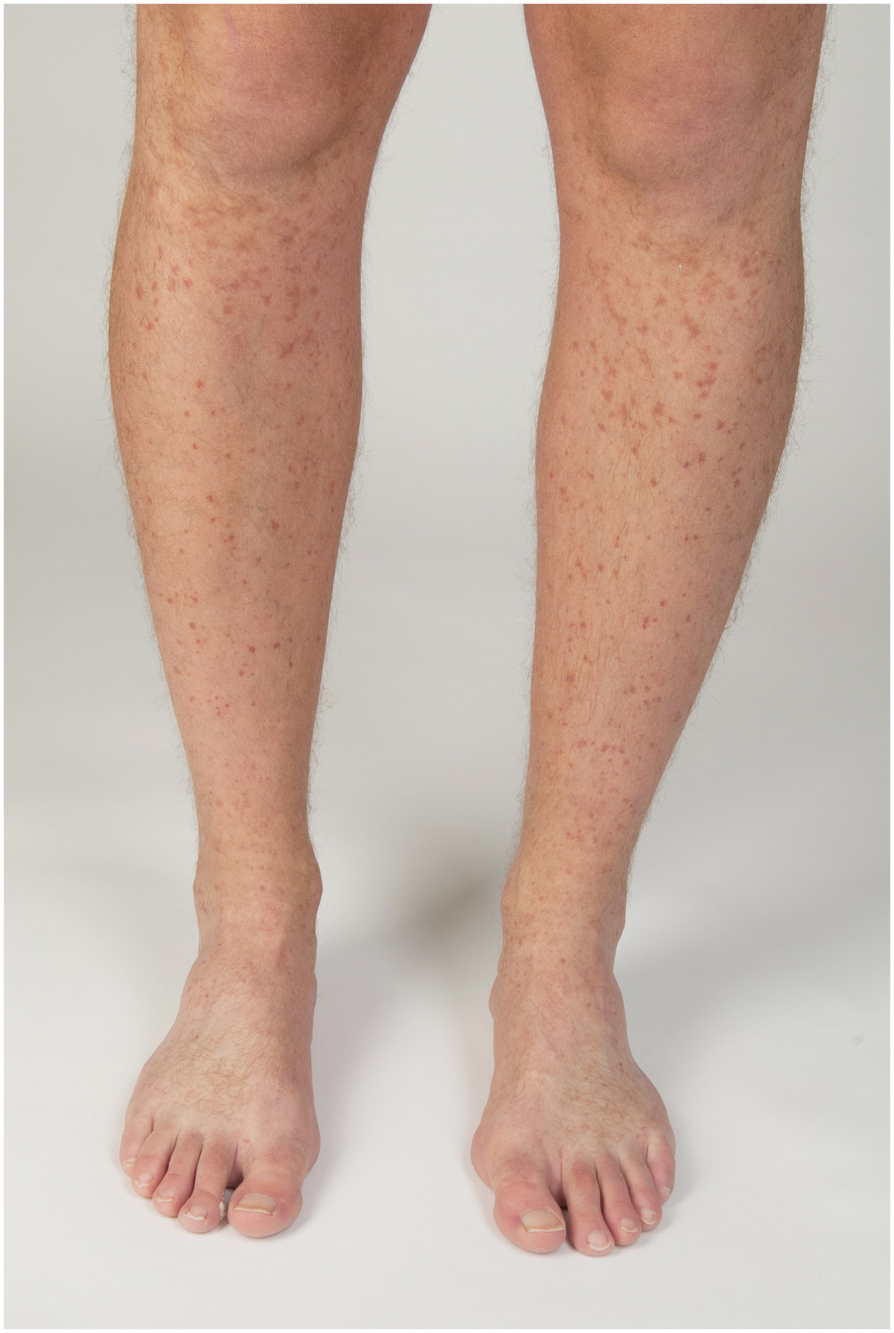
Recurrent macular vasculitis associated with hypergammaglobulinemia and induced by exertion.

There are different forms of immune complex vasculitides (Table 1) (1): systemic and skin-limited variants (1) of IgA vasculitis (IgAV) (Figure 1), cryoglobulinemic vasculitis (CV), rheumatoid vasculitis (RV), lupus vasculitis (LE vasculitis), hypocomplementemic vasculitis (Figure 2), and serum sickness, as well as the provisionally defined forms of cutaneous IgM/IgG immune complex vasculitides and a recurrent macular vasculitis induced by hypergammaglobulinemia (Waldenström) (Figure 3) or by exertion or heat, or a, vasculitis in gammopathy other than cryoglobulinemic vasculitis (2, 3).

**Table 1:** Immune complex vasculitides.

IgA vasculitis (IgAV)(Henoch-Schönlein purpura) - systemic IgAV - skin-limited IgAV - provisional: IgA nephropathy (IgAN) (if considered as kidney-limited IgAV)	Vasculitis, with immune deposits of hypogalactosidated IgA (Gd-IgA1), affecting small vessels (predominantly post-capillary venules)
IgM/IgG vasculitis (provisional, skin-limited)	Vasculitis, with IgM and/or IgG dominant immune deposits, not containing IgA, and independent of Gd-IgA1, affecting small vessels (predominantly post-capillary venules) in the skin
Cryoglobulinemic vasculitis (CV) - systemic CV - skin-limited CV	Vasculitis with cryoglobulin immune deposits, mostly IC, affecting small vessels and associated with serum cryoglobulins, usually type II or III
Vasculitis associated with systemic, usually collagenous vascular, disease: e.g., - rheumatoid vasculitis (RV) - LE vasculitis - systemic skin-limited forms of vasculitis	Vasculitis that is associated with and maybe secondary to (caused by) a systemic disease (e.g., rheumatoid vasculitis, LE, sarcoid vasculitis, etc.). The name (diagnosis) should have a prefix term specifying the systemic disease (e.g., rheumatoid vasculitis, lupus vasculitis, etc.)
Hypo-complementemic urticarial vasculitis (HUV) (anti-C1q vasculitis)	Vasculitis accompanied by urticarial lesions and hypo-complementemia affecting small vessels and associated with anti-C1q antibodies. Glomerulonephritis, arthritis, obstructive pulmonary disease, and ocular inflammation are common.
Hypo- or normocomplementemic urticarial vasculitis (non-anti-C1q) (provisional) (skin-limited)	Cutaneous, leukocytoclastic vasculitis, clinically appearing as urticarial lesions or wheals with hemorrhagic macules, affecting small vessels and not associated with anti-C1q antibodies (it is a provisional term); several previously published cases of so-called urticarial vasculitis may today be diagnosed as neutrophilic urticarial dermatosis (NUD) and not as vasculitis
Recurrent macular vasculitis in hypergammaglobulinemia (formerly benign hypergammaglobulinemic purpura of Waldenström) or recurrent macular vasculitis mediated by exertion (Golfer's vasculitis, cocktail party vasculitis, heat-induced vasculitis)	Relapsing, short-lasting cutaneous small vessel vasculitis with recurring macules and purpura associated with vascular immunoglobulin deposits and hypergammaglobulinemia or possibly vasodilation induced by exertion, alcohol, long standing, or heat

According to Coombs and Gell, the so-called type III hypersensitivity reaction has been considered among immunologists as a model for immune complex vasculitis. The prototype would have been that of serum sickness that occurs when a certain antigen in the circulation encounters approximately equimolar concentrations of fitting antibodies (4, 5) so that large lattices of circulating immune complexes form, which subsequently become deposited at the walls of small blood vessels, where they activate the complement system and polymorphonuclear neutrophils (PMNs).

This disease has has long been considered to present the prime example for immune complex vasculitis and corresponding animal models, including the Arthus reaction, and has largely dominated the pathophysiological concept of immune complex disease in general and immune complex vasculitis in particular.

However, the pathophysiology of IgA vasculitis has been revealed to differ at least in its initial stages from serum sickness, because it is primarily the altered galactosidation of the IgA1 molecule which mediates its deposition at certain vasculatures and not the size of the IC (6, 7).

Circulating complexes have also been demonstrated to present a major pathophysiological factor for vasculitis in cryoglobulinemia and in rheumatoid arthritis (RA) (1).

Common denominators for immune complex vascultides are perivascular deposition of altered immunoglobulins or immune complexes, and their subsequent full activation of PMNs close to the vessel wall *via* FcyR or FcαR. Histologically, the resulting picture of small vessel vasculitis is generally that of leukocytoclastic vasculitis of post-capillary venules, which sometimes extends into small veins and may also involve small arterioles. Clinically, the inflammatory infiltrates and ensuing extravasation of blood manifests as hemorrhagic maculae, papules, and plaques, sometimes in a retiform pattern [branched or retiform purpura, a term coined by Warren Piette (8)]. The most characteristic clinical picture as it has engraved in the clinician's eye is that presented by IgA vasculitis, i.e., palpable and macular, round or oval, and sometimes branched or retiform purpura with a predilection for dependent parts, namely the legs (Figure 1). In the other forms of immune complex vasculitis, the lesions are not as numerous and not as accentuated on the lower legs as in IgA vasculitis.

As reflected by the nomenclature of Chapel Hill Consensus Conference (CHCC) 2012 for cutaneous vasculitides, several of the different forms of systemic immune complex vasculitides have a counterpart that seems to occur exclusively on the skin but has the same cutaneous manifestations clinically and histologically as the systemic form. They are referred to as a skin-limited or cutaneous form of the respective systemic vasculitis (e.g., cutaneous IgA vasculitis) (1).

While most cutaneous vasculitides are immune complex-mediated vasculitides of mainly small vessels, there are several other vasculitides of small vessels (ANCA-associated vasculitides), medium (cutaneous periarteritis nodosa), and even large vessels (9) that affect the skin.

## 2. Serum sickness disease

Serum sickness disease is a paradigm of a systemic immune complex disease (type III hypersensitivity reaction). In its complete form, it apparently results when large amounts of heterologous (non-human) proteins as antigens encounter approximately equimolar concentrations of fitting antibodies leading to formation of large lattices of immune complexes. It induces not only IgG but also IgE, so serum sickness causes symptoms due to a varying involvement of the activated complement cascade and IgE, depending on the antigen. Histamine-mediated vascular permeability of vessels and joints facilitates perivascular deposition of ICs. Nowadays, serum sickness disease occurs not only after the administration of antithymocyte globulin (rabbit serum) but also after the administration of other foreign sera (antidotes), rituximab, streptokinase, or other drugs. However, one may speculate that there are attenuated or abortive forms with perhaps only skin-limited perivascular deposition of immune complexes, one of whose clinical manifestations could be cutaneous IgG/IgM vasculitis (refer to the following).

The typical symptoms are persistent fever, arthralgia or arthritis mostly of the large joints, lymphadenopathy, asthma, and a polymorphic picture with urticae, maculopapular exanthema, itchy papules, or palpable purpura. In contrast, IgA or IgG/IgM vasculitis would typically present on the skin with only macular or palpable round, oval, or retiform purpura with a predilection for the legs. Symptoms occur 7–14 days after primary administration (and 2–4 days after repeated administration) of a foreign protein. Accordingly, high levels of circulating immune complexes are detectable after 10–12 days and low levels of C4 and C3 on the 10th day, while C3a anaphylatoxin is elevated, accompanied by leukocytosis and sometimes eosinophilia, hematuria, and proteinuria.

## 3. IgA vasculitis

Immunoglobulins A vasculitis is an inflammation of small vessels related to tissue deposits of immunoglobulins A (IgA), or, more precisely, galactose-deficient or hypogalactosidated IgA1 (Gd IgA1), most likely bound in immune complexes. It may present as a variant restricted only to the skin (skin-limited IgAV) or as systemic vasculitis [IgAV–Henoch-Schönlein Purpura (HSP)], which manifests as arthritis, gastrointestinal vasculitis, renal vasculitis, and rarely pulmonary or cerebrovascular vasculitis. The reason why there is a skin-limited or a systemic form is not due to presence or absence of perivascular deposition of Gd-IgA1 because we detected Gd-IgA1 around cutaneous vessels in both systemic as well as skin-limited IgAV (10).

Similar to skin-limited IgAV, IgA nephropathy (IgAN), the most frequent form of nephritis in adults, may also present an organ-limited variant in the spectrum of IgA-related vasculitides, restricted to the kidneys. Histopathologically, IgA-nephritis in the course of systemic IgAV is indistinguishable from IgAN [for review (6, 7, 11, 12)]. There are, however, some differences, as the episode of kidney injury in IgAN is mainly chronic and presents with less crescentic lesions and more sclerotic lesions than in IgAV-nephritis; in addition, IgAV-nephritis shows more glomerular capillaritis with subendothelial IgA deposition and significant elevation of serum inflammatory cytokines (13).

The short-term prognosis of systemic IgAV depends on the severity of potential acute involvement of the gastrointestinal tract, while the long-term prognosis is dependent on the extent of the kidney damage. End-stage renal failure may occur more than 10 years after the onset of IgAV (14, 15).

### 3.1. IgA and Gd-IgA1

Immunoglobulins A presents an important immunoglobulin in mucosal immunity. It is produced by the B cells in the mucosal-associated lymphoid tissue and the bone marrow. The major part of serum IgA (90%) is IgA1. More than 90% of serum IgA1 is monomeric, while IgA1 secreted by mucosal tissues is mainly polymeric (linked by joining chains), a quality that is relevant for IgAV [for review (6)].

Hypoglycolysation occurs only in IgA1 and not in IgA2 because IgA1 has an extended hinge region with the insertion of two octapeptide repeats in its heavy chain. The repeats have three to six common O-glycan sites consisting of serine or threonine residues to which galactose or sialic acid bind. This binding is catalyzed by several transferases such as polypeptide N-acetylgalactosaminyl transferase 2 or core-1 β1,3-galactosyltransferase (which catalyzed the binding of Gal to GalNAc) and acetylgalactosamine-specific α-2,6 sialic acid transferase, respectively. Altered expression and activities of galactosyltransferase would result in altered exposure of terminal GalNac residues or more Gd-IgA [for review, refer to Heineke et al. (6), Barratt et al. (12), and Xu et al. (16)].

Gd-IgA1 is also found in healthy individuals, albeit in low concentrations [Suzuki et al. (17), for review: (12)]. In contrast, adult patients with IgAN and IgAV nephritis appear to have an inherited autosomal dominant or a constitutional disposition for elevated levels of Gd-IgA1 (12, 18, 19). In addition, immortalized B cells from patients with IgAV and IgAV nephritis both produced similarly high amounts of GdIgA1, while cell lines from patients with IgAV without nephritis produced mostly normally galactosidated IgA (17). Formation of Gd-IgA1 at least in IgAN is additionally enhanced by interleukin-6 (IL-6), interleukin-4 (IL-4), and even by some miRNA (miR-148b); their respective actions result in reduced activity of transferases or other reactions in glycosylation [for review, refer to Xu et al. (16)].

Most, but not all, studies have demonstrated elevated serum levels of Gd-IgA1 compared to controls in the majority of patients with IgAN [e.g., 75% of children with IgAN (20)] and also in patients with IgAV-nephritis [e.g., 52% of children with IgAV nephritis (20)]. In other studies [from France or in China (21, 22)], significantly higher serum levels of Gd-IgA1 were not only found when compared with healthy controls but also when distinguishing children or adults with IgAV nephritis from those with IgAV ***without*** nephritis. In contrast, other studies showed that serum levels did not significantly differ (i) between IgAV ***without*** nephritis (likely what we now call skin-limited IgAV) and healthy controls (12, 23) or (ii) between children with IgAV and children with inactive IgAV-nephritis or with controls (12).

### 3.2. Causes for increased serum levels

A major reason for the overall rise of IgA, IgA1, and Gd-IgA1 is probably the stimulation of B cells and their IgA production by infectious organisms or other agents.

In IgAN, one has several hints that dysregulation of lymphoid organs in the intestine may be the inciting event that leads to stimulation of GdIgA1 production [for review (7)], partially since (i) budesonide, a corticosteroid that acts exclusively in the gastrointestinal tract, has improved IgAN in phase 2 (NEFIGAN) and phase 3 (NEFIGARD) clinical trials (24, 25), (ii) microorganisms induce activation factors for B lymphocytes, (iii) certain compositions of the intestinal microbiota are associated with IgAN, and (iv) pan-genomic association genetic studies (GWAS) show an association between IgAN and genes involved in immunity to intestinal pathogens or in the maintenance of intestinal barrier [for review (7)].

In contrast, one has only circumstantial evidence that in IgAV, other mucosal surfaces and immune systems also are involved in the stimulation of IgA production. As such, IgAV has been observed to be often preceded by an infection of e.g., the upper digestive or respiratory tract (streptococcus, adenovirus, parvovirus, and *Mycoplasma pneumoniae*) or by systemic infection with Parvovirus B19, EBV, CMV, HIV, and COVID-19 [(26), for more literature (7, 12)], as well as by the intake of drugs, certain toxins, or food, especially in children.

Therefore, a situational rise in IgA1 together with a proportional or even disproportional increase in Gd-IgA1 (due to genetic predisposition in response to mostly mucosal infection and IL-6 production) is one incipient step in the pathophysiology of IgAV. The question remains if some of these probably eliciting agents are physically enclosed in immune complexes or facilitate the formation of, in particular, large immune complexes.

One further prerequisite besides elevated Gd-IgA1 levels appears to be indeed their binding in immune complexes. In patients with IgAN, but also in children with IgAV, IgG autoantibodies to Gd-IgA1 were found in IgAN (27) and in those children with IgAV nephritis (17). Patients with active IgAV nephritis showed higher serum levels of Gd-IgA1-specific IgG autoantibodies than patients with inactive IgAV-nephritis or than patients with IgAV ***without*** renal involvement whose levels rather were similar to healthy controls (17).

In the rarely performed serial determination of the levels of IgG autoantibodies, serum levels of IgG autoantibodies appear to correlate with serum levels of Gd-IgA1 in IgAN (28). IgG autoantibodies were found to be enriched in the glomerular immune deposits of kidney biopsies from patients with IgAN, but not those from patients with other forms of nephritis, i.e., lupus nephritis or membranous nephropathy (28).

In IgAV, levels of circulating IgA immune complexes were significantly correlated with the detection of IgA in kidneys (29) and with the presence of signs of clinical and histological activity. These signs were the magnitude of microscopic hematuria, a past history of macroscopic hematuria, and the percentage of glomeruli with florid epithelial crescents [(30), for further literature, refer to (16)].

In addition, the size of immune complexes appears to be decisive for the occurrence of IgAN or IgAV nephritis. While all patients with IgAV had circulating IgA1-containing ICs of a relatively small molecular mass, patients with IgAV nephritis had additional large-molecular mass IgA1–IgG immune complexes. This is in line with our observation that only large ICs or aggregated IgA would deposit on vessel walls and elicit ensuing reactions (31).

### 3.3. Perivascular and mesangial deposition of Gd-IgA1

The reason for the perivascular and mesangial deposition of IgA or GdIgA1 apparently has to do with the reduced glycosylation of IgA1, which modifies the binding affinity to its receptors, (i) the RFcαI (CD89) on circulating monocytes and neutrophils, and (ii) the transferrin receptor (CD71) on mesangial cells. Moreover, the binding of abnormally glycosylated IgA molecules to CD89 induces its release into the circulation, so that blood contains circulating complexes of soluble CD89 and IgA in both IgAV nephritis and IgAN (21, 23).

While the deposition of this complex in the renal mesangium is facilitated by binding to CD71, which is supposed to be even overexpressed by mesangial cells in patients with IgAN, the reason for its deposition in post-capillary venules of the skin is less clear. Endothelial cells (EC) have been shown to express FcyR (32), and while FcRαI (CD89) and CD71 are not explicitly listed as receptors of EC (33), CD71 is known to be expressed by most cells, albeit in low levels, and there are several additional IgA receptors. Thus, one could speculate (i) that IgA is bound by so far unidentified receptors on dermal EC, but perhaps not only when IgA levels in serum are high or (ii) that it may become physically trapped between EC and pericytes, especially when it is part of a larger complex (e.g., IgA sCD89 or IgA–IgG) and when there are vasodilatory gaps between EC. Such larger molecules in the blood may drift to the vascular wall when blood flow slows down considerably in the vascular beds of post-capillary venules (according to the model of laminar, parabolic flow) or when blood flow becomes partially turbulent (according to newer models on blood flow). This resembles the concept proposed for large complexes and their perivascular deposition in serum sickness (34). The predilection at lower legs where blood flow is supposed to slow down due to (physiological) stasis further supports the relevance of reduced blood velocity for perivascular depositions. An additional factor could be the activation of EC because we observed *in vitro* that IgA complexes adhere more readily to EC (HUVEC) when they are activated (31). The molecular basis for this observation is not known.

It remains noteworthy that in all these studies, not all patients with active disease presented elevated levels of IgA and, in particular, of Gd-IgA1 or sCD89-Gd-IgA1. One can speculate that it has to do with only a transient rise, which was no longer detectable at the time of blood sampling. Yet, the presence of elevated serum Gd-IgA1 levels alone still would not result in IgAV nephritis or IgAN. This is underlined by the fact that high serum levels are inherited in pediatric patients with IgAN and IgAV nephritis, but their first-degree relatives who also had elevated serum Gd-IgA1 levels never had clinical features of IgAN or IgAV (18, 19).

Similarly, glomerular Gd-IgA1 deposition is not specific for IgAN and IgAV nephritis, but may also occur in IgAN with hepatitis B virus antigen or in lupus nephritis [only that their amount is higher in IgAN, and glomerular IgG seems indeed specific for Gd-IgA1 (35)].

### 3.4. Activation of PMNs and destruction of vessel walls

The deposition of immune complexes in the vascular wall is one major igniting factor of the local vasculitic reaction, mainly by activating PMNs (8, 36). *In vitro*, IgG (37–39) and IgA (40), when fixed in the solid phase, activate PMNs to undergo oxidative burst, degranulation, and NETosis. However, deposits are also found in clinically and histologically normal skin between or after flares of vasculitis (10), so perivascular deposition of IgA, IgG, and IgM is mandatory, but not sufficient for eliciting vasculitis in the tissue.

We demonstrated that during episodes of active IgAV, circulating PMNs additionally bind and are primed by circulating IgA complexes. The IgA complexes were elevated in serum during these intervals. This binding of IgA to circulating PMNs greatly amplified two ensuing processes critical for local vessel destruction, i.e., (i) firm and continuous adherence of PMNs to the wall of post-capillary venules with deposited IgA, and (ii) release of high amounts of cytotoxic NETs in proximity to the vessel wall. This priming only occurred after the binding of large polymeric IgA molecules or of IgA-immune complexes, but not in presence of monomeric IgA (which normally is more prevalent in the blood). Priming is mediated by (crosslinking of) FcαRI (CD89) (31). When FcαRI is cross-linked by IgA-immune complexes or by aggregated IgA, it can form complexes with the FcR γ chain, which contains the “Immunoreceptor Tyrosine-based Activation Motifs” (ITAMs). This way it propagates downstream signals and activates neutrophils for pro-inflammatory functions, such as phagocytosis, production of reactive oxygen species (ROS), NETosis, and release of cytokines or chemokines. In contrast, monomeric IgA can bind to, but not cross-link, FcαRI. The binding of only a single FcαRI subsequently induces anti-inflammatory responses, because monovalent targeting of FcαRI results in the formation of “inhibisomes,” which impair the signaling of neighboring activated receptors [for review, refer to (6)].

These processes were not encountered in other non-vasculitic inflammations such as psoriasis.

Polymorphonuclear neutrophils can also be primed by IL6, which is one of the cytokines found to be elevated in patients with IgAV.

Immunoglobulins A-binding PMNs from patients with IgAV even show spontaneous NETosis in static *in vitro* assay. Such a lower threshold to undergo spontaneous NETosis has been reported only rarely, such as in ANCA-associated vasculitis (AAV) (41, 42), systemic lupus erythematosus (43), and in the context of SARS-CoV-2 infection (44). Yet, in IgAV, it did not become meaningful *in vivo*, unless PMNs had adhered to EC, explaining why NETosis is not observed in the circulation *in vivo* in IgAV or other systemic diseases (31).

In the skin, IgAV and other IC-mediated vasculitides occur in the post-capillary venules, notably the site of leucocyte transmigration, where damaging events start at the luminal aspect of the vessel (45). One would expect that cytotoxic reagents would rapidly be spilled away by the bloodstream, but (i) blow flow is considered to be very slow at the wall of vessels and (ii) binding of IgA-immune complexes to circulating PMNs promotes and augments PMN adherence to ECs. PMN have been shown to cause damage to ECs by NETosis in static *in vitro* assays (46, 47). We recently demonstrated that under flow conditions in a perfusion system, NETs, instead of floating freely or flowing away, co-localized spatially and temporally with the site of damage in the EC layer. Correspondingly, we were able to visualize *in vivo* in vasculitic lesions that NET proteins were located on the luminal side of post-capillary venules and associated with damaged blood vessels in incipient lesions of IgAV (31).

In summary, for marked NETosis to occur, as well as for oxidative burst and degranulation (37, 48), PMNs require both exposure to IgA-IC in the circulation and adherence to ECs, which, however, then occurs so close to endothelial layers that it results in damage.

With regard to complement, PMN activation in the skin is ***not*** dependent on the activation of complement. This holds true although IgA aggregates can activate complement *in vitro via* alternative pathway and *via* the lectin pathway carbohydrate recognition molecule, MBL (49). In contrast, activation of complement appears to be mandatory in the kidneys for the pathophysiology of IgAN (49) or even IgAV with nephritis (50). Deposits of C3 and other components of complement are seen deposited around some cutaneous vessels in IgAV, but not as regularly as in kidneys, and are not *per se* signs of complement activation [reviewed in Damman et al. (50)].

The tissue-specific microenvironment, the complex glycocalyx of the kidney [which has influence on complement regulation (51)], combined with genetic differences in complement genes (49), may offer one clue: why in the spectrum of IgA-immune complex diseases we see either isolated IgAN or skin-limited IgAV, or both.

### 3.5. A possible sequence of events

We suggest the following sequence of events for the development of cutaneous, and possibly systemic, IgAV lesions (31): 1 general stimulation of B cells due to, e.g., infection or drug intake in patients with constitutional production of GdIgA; 2 intermittently raised levels of both GdIgA and IgG autoantibodies directed against GdIgA; 3 formation of circulating IgA-immune complexes of GdIgA1 with IgG antibodies or soluble CD89 or of aggregated Gd-IgA whose size surpasses a certain threshold to enable cross binding of IgA receptors on PMNs and deposition at vascular wall; 4 binding of IgA-immune complexes to PMNs in the circulation (to a much larger extent than in healthy individuals or even in other pathological conditions); 5 pre-stimulation of PMNs, lowering the threshold for NETosis, but not eliciting NETosis yet without adhesion of PMNs; 6 additional, albeit minor, PMN prestimulation also occurs through exposure to cytokines which are elevated in IgAV, e.g., IL-6; 7 activation of EC with expression of adhesion molecules, vasodilation, IgA receptor molecules on EC, the higher serum levels, or large size of immune complexes facilitate deposition of immune complexes at vessel walls; 8 IgA-bearing PMNs firmly adhere to EC; resulting in 9 complete PMN activation and marked release of NETs; which 10 anchor to the luminal side of the EC layer without being cleared by the blood stream; and 11 cause destruction of the post-capillary venule walls. This concept still entails some as far unresolved steps but would explain why IgA-immune complexes are found around blood vessels for some time (and likely also in kidneys and other organs) without causing tissue damage.

The sequence of events in the kidney and other organs is similar [reviewed, e.g., by Pillebout and Sunderkotter (7)], but differs in certain respects, e.g., in the steady colocalization of C3 with IgA1 deposits and the necessity for activation of complement.

Animal models, which usually are run with IgG-containing immune complexes, have lead to similar concepts, i.e., that the deposition of ICs in the vascular wall is the major igniting factor of the inflammatory cascade (7), mainly by activating PMNs (8). *In vitro* studies support this proposition, as IgG (9–11), similar to IgA (12), activates PMNs to undergo oxidative burst, degranulation, and NETosis when fixed in the solid phase.

## 4. IgG-/IgM-positive (IgA-negative) immune complex vasculitis

If no IgA, but IgG or IgM, is detected around vessels, this indicates another subtype of immune complex vasculitis, e.g., cryoglobulinemic vasculitis, hypocomplementaemic vasculitis (Figure 2), or systemic disease associated with IC-mediated vasculitis such as rheumatoid arthritis or vasculitis in SLE (Table 1).

Primary or genuine IgG-/IgM-vasculitis (i.e., IgG-/IgM-vasculitis without an underlying systemic disease which is associated with dysregulation of B cells or antibodies), analogous to IgA1-vasculitis, is much rarer than originally assumed. It was mostly meant in the literature and in clinical practice when the terms hypersensitivity vasculitis or leukocytoclastic vasculitis were used. Yet, while many dermatologists are convinced that it exists and that it presents an entity of its own, there are only a few published studies so far which would confirm it (52). Its underlying cause would be the deposition of immune complexes containing IgG or IgM, but no IgA. It may, thus, present a form of a—likely skin-limited—serum sickness reaction. The reason for perivascular deposition of immune complexes in these cases would then be their sheer size (presumably not altered galactosidation). Their larger size might propel them to the edge of vessels where blood flow is much slower (according to the model of laminar, parabolic flow) or also partially turbulent (according to newer models on blood flow). The altered flow or markedly reduced velocity of blood and particles along the vessel wall and the size of the IC may facilitate their entrapment between EC and pericytes (53). Marginating PMNs, which are in the process of adhering to endothelial cells and of transmigration, will also bind to the IgG/IgM complexes *via* their FcyR. Once PMNs are adherent, they become more easily and more vigorously activated after their FcyR are crosslinked by IgG/IgM complexes (36, 37, 48). The ensuing release of cytotoxic products would subsequently occur close to the vessel wall, perhaps during the diapedesis of PMNs, thus damaging the vessel, a scenario long described and ultrastructurally shown in the Arthus reaction in animals (54).

We learned from IgA vasculitis that this scenario may contain some oversimplifications. Clinically, it would feature palpable and retiform purpura as in IgAV.

## 5. Recurrent macular vasculitis in hypergammaglobulinemia (formerly called benign hypergammaglobulinemic purpura of Waldenström) or mediated by exertion (Golfer's vasculitis, cocktail party vasculitis, and heat-induced vasculitis)

Recurrent macular vasculitis in hypergammaglobulinemia is a chronic episodic vasculitis of small blood vessels with vascular deposits of immunoglobulins, often associated with (a) a hypergammglobulinemia (usually polyclonal, but sometimes also monoclonal) and an elevated sedimentation rate and/or (b) induction by vasodilatory or stasis-associated events, such as long-standing and consumption of alcohol (cocktail party), or playing golf, extended hikes in association with warm weather, or other kinds of exertion (thus the different names for it). A characteristic feature is the chronic relapsing sudden occurrence of many (>50) small short-lived hemorrhagic macules on lower legs which hardly leave any traces except occasionally slight macular hyperpigmentation. In contrast to other immune complex-related vasculitides, these lesions are regularly associated with a burning sensation. Since biopsies often, but not regularly, reveal perivascular deposits of IgG or also IgA, the clinical picture may putatively be due to a very transient vascular deposition of immune complexes. In several, but not all patients, IgG or IgA rheumatoid factor (RF) is detected in serum, which is highly soluble and could therefore resolve rapidly after vascular deposition (55). Therefore, vascular damage may be subtle and quickly reversible, thus leading to leakage of red blood cells but not to full, irreversible destruction of vessel walls.

## 6. Cryoglobulinemic vasculitis (CV)

Cryoglobulinemic vasculitis is a leukocytoclastic immune complex vasculitis seen in type II, less often in type III, hypersensitivity mixed cryoglobulinemia. In rare cases, it has been described with type I monoclonal cryoglobulinemia, which, however, are not immune complexes *per se* (56, 57); it needs to be carefully clarified if it exists independently from gelling of type I cryoglobulins (similarly in vasculitis associated with monoclonal gammopathy) (58).

Cryoglobulins are immunoglobulins that precipitate *in vitro* at temperatures below 37°C (*in vivo* depending on pH, ion concentration, high content of hydrophobic amino acids, low content of tyrosine residues, or galactose and sialic acid). Types II and III are immune complexes because in type II cryoglobulinemia, monoclonal IgM forms a complex with IgG. In many cases, this IgG is directed against the non-enveloped core protein of the hepatitis C virus and can then precipitate *via* a conformational change in the complex at cold temperatures.

In type I cryoglobulinemia, the monoclonal cryoglobulins gel directly in cold and in the presence of one of the above factors, causing hyperviscosity and subsequently vascular occlusion and ischemic necrosis.

Both features may occur simultaneously because the i) slowing of blood flow, cooling, and gelation, as well as ii) the deposition of immune complexes, influence and reinforce each other. Thus, in rare cases, mixed cryoglobulinemia also leads to hyperviscosity (< 3%), and slowing down of blood flow with ensuing deposition of immune complexes and cryoglobulinemia type I result in vasculitis (57).

This vasculitis mainly affects small vessels and can also include medium-sized and even large-sized vessels (aorta and branches). Vasculitis usually involves the skin in the systemic form, but there is a skin-limited form without the involvement of visceral organs.

Thus, the involvement of skin in cryoglobulinemia is due to two major mechanisms of tissue damage: (i) leukocytoclastic immune complex vasculitis (with the formation of cryoglobulins being primarily unrelated to cold) and/or (ii) occlusion of cutaneous vessels by gelling or precipitation of mostly monoclonal type I cryoglobulins in cold-exposed skin areas (all small blood vessels of the upper or deep dermis, as well as the capillaries of the fat lobule, may be involved) (vasculopathy) (59).

Clinically, leukocytoclastic immune complex-mediated vasculitis manifests as palpable or retiform purpura lesions that may coalesce. Occlusion of vessels by cryoglobulins manifests clinically as retiform purpura with dominant central necrosis (larger than the surrounding inflammatory erythema) in cold-exposed, acral areas (hands, feet, lips, ears, and nose), sometimes accompanied by livedo due to only partial obstruction of blood flow.

## 7. Rheumatoid vasculitis, lupus vasculitis, and Sjögren's syndrome

Rheumatoid vasculitis (RV) is a severe complication of **rheumatoid arthritis** (RA), characterized by cutaneous and systemic vasculitis affecting small or medium-sized vessels and occurring usually in patients who had high titers of RF over a long period of time. Other factors associated with the development of RV were male gender, joint erosions, subcutaneous nodules, presence of nail fold lesions, and any other extra-articular feature 1 year before the time of diagnosis of RV and intensive treatment with antirheumatic drugs (60).

Rheumatoid factor is an immune complex. Circulating RF contain IgG and IgA (61). Decreased C3 complement levels indicate marked activation of the complement system. Although there is not much recent research on RV, all facts known so far indicate that it is an immune complex vasculitis owed to similar mechanisms as in cryoglobulinemic vasculitis or the Arthus reaction.

Since cutaneous vasculitis in rheumatoid vasculitis ranges from leukocytoclastic vasculitis of post-capillary venules to arteritis located at the dermo-subcutaneous junction or in the panniculus (62), it clinically reveals a spectrum from palpable purpura as in IgAV or IgG/IgM vasculitis to livedo reticularis (racemosa) and ulcerating nodules similar to cutaneous arteritis, or even digital infarcts and gangrene (62).

Cutaneous vasculitis in **Lupus erythematodes** often occurs as small vessel vasculitis with perivascular IgG deposits, or as so-called hypocomplementemic (urticarial) vasculitis with or without C1q antibodies. It likely is the result of several pathomechanisms, but encompassing immune complexes.

Vasculitis in **Sjögren's syndrome** is often either r*ecurrent macular vasculitis in hypergammaglobulinemia* (as mentioned earlier) *or* cryoglobulinemic vasculitis or vasculopathy. In the former high titers of small circulating immune complexes containing IgG or IgA, RF has been detected in some but not all cases as part of the gammaglobulin fraction (55). Cryoglobulinemic vasculitis in Sjögren's syndrome bears a risk for lymphoma (57, 63), whereas recurrent macular vasculitis in hypergammaglobulinemia does not share this risk (63).

## 8. Concluding remarks

Although several steps in the pathophysiology of the different immune complex vasculitides have been elucidated, several remain to be explored; some of them emerging from the fog in the form of already deducted hypotheses that wait to be verified or falsified, while others remain in the dark. Shrouded in the mist are the steps to target efficacious therapies, but in this field, light may be ahead at least for some small steps because budesonide or antibodies to complement components showed effects in first clinical trials.

## Author contributions

CS contributed to the conception of the work and drafting of the work. LG contributed to the design of the work, drafting of the work, revising it critically for important intellectual content, and provide approval for publication of the content. EP and CM contributed to the conception of the work, drafting of the work, and revising it critically for important intellectual content. All authors contributed to the article and approved the submitted version.
